# Industrial upgrading and its influence on green land use efficiency

**DOI:** 10.1038/s41598-023-29928-8

**Published:** 2023-02-16

**Authors:** Jifeng Chang, Wei Wang, Jinli Liu

**Affiliations:** 1 School of Finance and Public Administration, Harbin University of Commerce, Harbin, Heilongjiang China; 2 School of Finance, Harbin University of Commerce, Harbin, Heilongjiang China

**Keywords:** Environmental sciences, Environmental social sciences, Energy science and technology

## Abstract

The rational use of land is an important guarantee for the sustainable development of resource-based cities. First, this paper uses the panel data of 115 resource-based cities in China from 2004 to 2018 to measure green land use efficiency (GLUE) by SBM model and Metafrontier-Malmquist productivity index model. Secondly, it analyzes the industrial upgrading from the perspectives of the optimization of industrial structure and the rationalization of industrial structure to explore the impact mechanism of industrial upgrading on GLUE. Furtherly, the heterogeneity analysis is carried out from the following two perspectives: exploring the impact of industrial upgrading on GLUE under different types of urban samples; using the quantile model to analyze the impact of industrial upgrading under different GLUE quantiles. The main conclusions of this study are as follows: (1) both the optimization of industrial structure and the rationalization of industrial structure promote the improvement of GLUE. The robustness test and instrumental variable method support this conclusion. (2) For different types of resource-based cities, the rationalization and optimization of industrial structure have positive coefficients in regeneration cities, growing cities, and declining cities, but have a negative effect on mature cities. (3) In all quantiles, the effects of industrial structure optimization and industrial structure rationalization are positive. However, with the increase of quantiles, the role of industrial structure optimization gradually decreases. The role of industrial structure rationalization gradually increases. Meanwhile, there is an obvious threshold effect when taking resource endowment as the threshold variable. This paper provides a theoretical reference for the transformation and development of resource-based cities.

## Introduction

With the development of China’s economy from “high speed” to “high quality”, the urbanization rate has increased from 17.9% in 1978 to 60.6% in 2019. However, as the main carrier of urban space, land use efficiency has not been significantly improved with the rapid development of urbanization. In addition, restricted by the policy of “arable land red line” and urban planning, the index of government construction land is gradually decreasing. The supply of urban construction land is becoming increasingly tight^1^. In the face of the contradiction between the increasing demand for land and the limited supply of land in urban development, it is important to improve land use efficiency^2^. Compared with cities with higher tertiary agglomeration, resource-based cities in China are facing more serious land predicament. Resource-based cities are mainly cities dominated by the exploitation and processing of natural resources (such as minerals and forests)^3,4^. The rise and fall of resource-based cities are closely related to the characteristics of natural resource endowments. The exploitation of resources has brought great economic benefits, but it has also led to the rough utilization of land resources. In addition to residential land and infrastructure construction land, industrial land is the main land for construction in resource-based cities^5^^,^^6^. In summary, resource-based cities are prone to low output efficiency and high consumption of land resources in the process of land use. Therefore, land has become a vital resource to solve the bottleneck development of resource-based cities^7^. Exploring the current situation and influencing factors of GLUE in resource-based cities not only help Chinese cities to achieve "high-quality" development, but also provide a reference for the development of resource-based cities in other countries^8^.

Existing studies have studied how to improve land use efficiency, such as economic growth^9^, technological progress^10^, demographic characteristics^10^, market reform^11^, government regulation^12^ and other factors have been proved to have an impact on land use. However, existing studies have ignored that industrial structure is a crucial factor leading to low land use efficiency in resource-based cities. When resource-based cities are faced with the predicament of resource exhaustion, it will inevitably lead to the migration of factories and the loss of labor. The original plant, mining area and other urban land often face the end of waste. For example, Daqing City, the largest oil city in China, whose total oil production accounts for 47% of the national land in the same period. It is known as “the miracle of world oil development history”. However, as the oil resources on which it depends have been exploited, oil production has dropped from 50 million tons in the peak period to 40 million tons. From 2014 to 2017, the population density of every square kilometer of land in Daqing City decreased by about 450 people. The outflow of population and industry has let to phenomenon of “ghost city” in cities. if there is no tertiary industry to reuse these lands after the outflow of resource-based industries, GLUE will decrease^13^. In addition, industrial upgrading can bring about the development of high-tech industries and realize the rational utilization of resources^14,15^. This can better solve the problems of industrial solidification^16^ and path dependence^17^ in resource-based cities. Therefore, the change of industrial structure has promoted the transformation of land nature from industrial land to commercial land, and injected new vitality into land use. Industrial upgrading can balance the relationship between economic development and resource utilization, optimize the industrial pattern, and thus improve the efficiency of land green utilization. However, there is no in-depth research on the role of industrial structure changes in land green utilization.

In view of this, this paper firstly uses the Metafrontier-Malmquist Productivity Index based on the SBM model to measure the GLUE of 115 resource-based cities in China. Secondly, represented by the optimization and rationalization of industrial structure, this paper uses panel data model to empirically test the impact of industrial structure upgrading on GLUE. Finally, considering the differences in the spatial distribution of GLUE, this paper uses the panel quantile model to empirically investigate the heterogeneous impact of industrial structure upgrading on GLUE.

This study may have the following contributions: (1) this paper first analyzes the important impact of industrial upgrading on the green utilization of resource-based urban land from both theoretical and empirical aspects, and conducts in-depth discussions from two aspects of industrial structure rationalization and industrial structure optimization. (2) The model uses the combination of SBM Model and Metafrontier-Malmquist Productivity Index Model, introduces the comprehensive pollution index of undesired output, measures the green use efficiency of land, combines quantile regression and threshold effect to discuss the efficiency of green land use at different levels in stages, revealing that industrial upgrading is different in different ways. The mechanism of action of stages on green land use efficiency. (3) For the first time, this paper uses the panel data of 115 resource-based cities in China from 2004 to 2018 to analyze the impact of industrial upgrading on the green utilization efficiency of land in resource-based cities.

**Figure 1:**
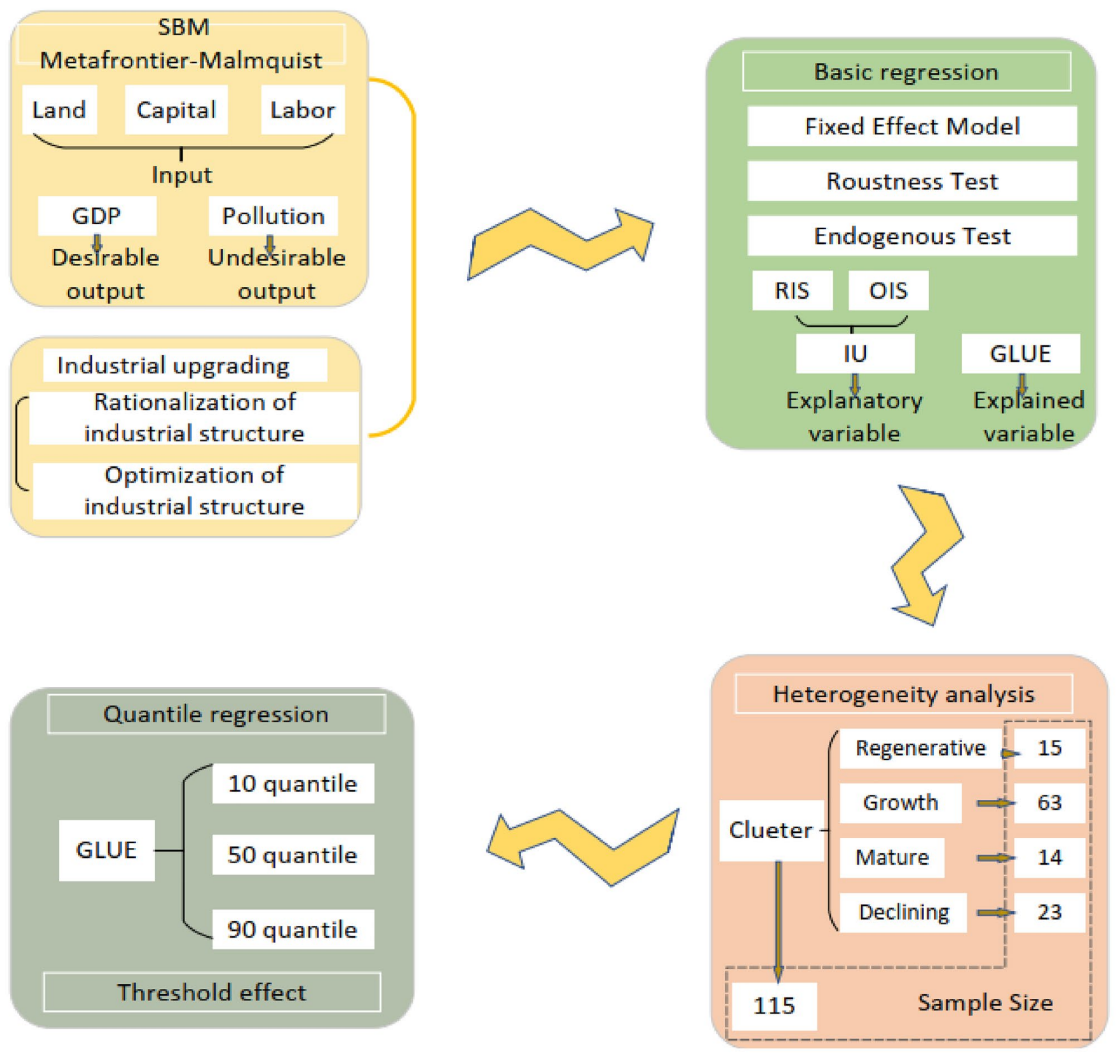
Analysis framework.

The second part of this paper makes a theoretical analysis of industrial structure upgrading and utilization efficiency of land. The third part proposes research hypothesis. The fourth part introduces the selection and model of variables. The fifth part includes panel regression analysis, quantile regression analysis and threshold effect. Meanwhile, further analyzes the relationship between industrial structure upgrading of different types of resource-based city and GLUE, and uses instrumental variable model and robustness test to support the conclusion. The sixth part conducts a discussion. The seventh part draws conclusions and policy recommendations. The analysis framework is shown in Fig. 1.

## Literature review

In the 1920s, resource-based cities attracted much attention from scholars. Auty^18^ put forward the concept of “resource curse”, which means countries or regions that abundant in natural resources develop more slowly than countries or regions scarce in natural resources, and resource wealth cannot be fully utilized, resulting in dependence on natural resources. It is obvious that such dependence leads to crowding out effect on other factors of production and difficulties in industrial transformation, Dutch diseases and crowding out effects^19–23^. When resource-based cities face the threat of “urban disease”, the problem, such as supply and demand structure imbalanced, supply mismatch, low-end overcapacity and other issues, are particularly serious, it should note that land is the main resource of economic development, and the problem of unreasonable use of land resources needs to be solved urgently.

The utilization of land plays an important role in economic development. Ricardo and Feng Duneng, two pioneers of discipline, put forward their own views. Ricardo believed that, with other conditions unchanged, the difference in rent between two lands should be equal to the difference in income between two lands.cc and Ricardo have the same point of view, and the theory is further embedded in the farmland model. Land use appears urban transportation, urban employment population problems and other modernization problems. In the process of development^24^, the transformation of land from natural state to human use has become the most basic and lasting effect in the interaction between human beings and natural resources. The classification of land specialization, the type and intensity of land development affect the transformation and strategic change of economic development. Therefore, the calculation and analysis of land use efficiency has important theoretical and practical significance. The utilization efficiency of land is perceived by applying urban economics, land economics, land use planning and other behavior planning. At first, the initial definition of land economics was limited to the perspective of economics. Stull^25^ proposed that land use is the premise of the optimal social and economic benefits. Subsequently, Eric^26^ put forward that when measuring the efficiency of land use, it should not only consider its economic implications, but also consider the political and social point of view. It enriched the principles of land use efficiency. In recent years, the problems of land use efficiency have attracted the attention of many scholars. In terms of the measurement of land use efficiency, the utilization efficiency of land uses a single element resource efficiency alternative method^27^, such as the use of unit area labor, capital and other input indicators as a substitute^28^. There are also scholars who use output indicators per unit of unit economic output value to replace^7^. However, the utilization efficiency is a complex process, single variable model cannot be fully reflected. Taking the social, economic, environmental and other multiple indicators into account, it is necessary to comprehensively consider the overall utilization efficiency of land. In terms of multi-factor research methods, some scholars used parameter measurement method to measure land use efficiency. For example, Liu et al.^29^ used SFA method to measure the GLUE in China and analyzed its improvement potential. Liu et al.^30^ used SFA method to measure China's industrial land efficiency and explored its influencing factors. The SFA method requires accurate selection of error term distribution and correct setting of production function form, which makes its application more difficult^11^. Due to this consideration, more and more scholars use DEA model to measure GLUE. Yu et al.^31^ used the combination methods of DEA model and Tapio model, from the perspective of efficiency evaluation, group analysis, efficiency decomposition and driving force analysis to evaluate. Compared with SFA method, DEA model does not need to set a specific function form. However, traditional DEA model is subject to static constraints, ignoring the existence of relaxation variables. Xie et al.^32^ used the global generalized directional distance function and DEA-malmquist index to explore the dynamic change of green land utilization rate in four major industrial parks in China. By using the super efficiency SBM model, Zhu et al.^33^ explored the spatial and temporal differences of GLUE in China’s mega-cities. The model improved the recognition and solved the nonlinear problem of the model under the condition of large sample size. This paper uses the Meta-frontier model based on SBM model to measure the GLUE of resource-based cities in China. Compared with other models, this model not only considers the undesired output, but also accurately reflects the dynamic changes of GLUE.

In terms of influencing factors, the degree of economic development^34^, policy changes^35^, the level of science and technology and industrial structure are the main factors affecting land use efficiency^36^. The upgrading of industrial structure is a dynamic process based on the characteristics of regional resources, matching with the internal and external environment of industrial development, constantly improving and upgrading the internal structure and continuously improving the overall efficiency of the industry. Yu et al.^31^ pointed out that when the industry expands and develops from the present stage to the industry with higher capital intensity, social demand^37^, technological innovation^38^, foreign trade^39^ and reform and innovation^40^ would promote the more rational use of resources. The spatial layout of land has been improved, resulting in the improvement of land use efficiency. In the measurement of industrial upgrading, previous studies only put the ratio of capital to labor force or the ratio of manufacturing to GDP to express the industrial structure^41^^,^^42^. However, these indicators only consider unilateral effects, which may lead to deviations in the results. Han et al.^43^ used industrial rationalization and industrial replacement to represent the upgrading of industrial structure through the evolutionary process of industrial balance, coordination and industrial structure from low to high.

In the study of the relationship between industrial structure and land use, different scholars come to different conclusions due to the different samples and models used. Some scholars have found that industrial structure can promote the efficient use of land. Dong et al.^44^ studied 108 cities in the Yangtze River Economic Belt generalized matrix method and pulse response. The study found that the core price index of urban agglomerations decreased accumulatively. Low-value areas spread from the city center to the surrounding area and found that industrial transformation and land use had a positive driving role. Chen et al.^34^ used Tobit model to analyze the influencing factors of industrial land efficiency in resource-based cities and found that industrial structure had a positive impact on industrial land use efficiency. Wang et al.^45^ empirically analyzed the impact of industrial agglomeration on urban land use efficiency by using the spatial panel model and found that industrial agglomeration could promote the improvement of urban land use efficiency. Some scholars have found that industrial structure has a negative impact on land use. Through the method of generalized least square and spatial measurement of provincial panel data from 2006 to 2018, Xu and Tan^46^ found that the industrial structure and the efficiency of natural resources utilization had a reverse effect on the low significant level and the elastic coefficient. Taking the panel data of 30 provinces in China from 2006 to 2020 as research samples, Li et al.^4^ empirically analyzed the impact of industrial structure optimization on the efficiency of industrial green land using the spatial Durbin model. It is found that the optimization of industrial structure has a negative impact on the efficiency of industrial green land, which mainly comes from the inefficiency and large-scale expansion of industrial land. Some scholars have found that there is a nonlinear relationship between industrial structure and land use. Taking the panel data of 31 provinces in China from 2000 to 2015 as research samples, Jingming Liu 2021 analyzed the impact of industrial structure optimization on urban land use efficiency by using the STIRPAT Model and the Spatial Durbin model. It is found that there is a U-shaped relationship between the optimization of industrial structure and urban land use efficiency.

In summary, although scholars have carried out extensive research on land use efficiency caused by industrial transformation and upgrading, the following aspects still need to be improved. (1) Most of the literatures ignore the internal analysis of industrial upgrading, and do not further explore industrial upgrading. (2) Scholars have studied more on the coordination, causality and linear relationship between industrial upgrading and land use efficiency, but less on the nonlinear relationship between industrial upgrading and different levels of GLUE. (3) The research of Scholars mainly focuses on the analysis of provincial data, ignoring the particularity of resource-based cities, which is not conducive to clarifying the real mechanism of green land use efficiency.

## Theoretical mechanisms and research hypotheses

GLUE refers to obtaining more economic benefits, social benefits and ecological benefits under the constraint of land resources to achieve coordinated development of economy, society and environment^47^. Compared with the general land use efficiency, it not only includes the economic value of land output, but also includes the social and environmental comprehensive value. Its core is to pursue the coordination and unity of social and economic development and ecological environment protection in urban land use system. The relationship between industrial upgrading and GLUE is mainly reflected in the impact of the upgrading and rationalization of industrial structure on GLUE.

The rationalization of industrial structure mainly affects the GLUE by producing “crowding out effect” and “substitution effect”. In terms of “crowding out effect”, “crowding out effect” refers to the government through the introduction of market mechanisms to screen the land user under the background of inelastic land supply. High pollution and low value-added value industries will be squeezed out of the market^48^. The surrounding areas will undertake the industrial transfer of the region to form industrial interaction. Low-pollution and high-value-added industries will be allocated to land with better geographical advantages. Meanwhile, influenced by the spillover of advanced knowledge and technology, the surrounding areas will accelerate the adjustment of industrial structure to realize the green and rational utilization of land resources. In terms of “substitution effect”, “substitution effect” is that the manufacturer will reset the quota of production factor input driven by the maximum marginal benefit. When the cost of land use is high, manufacturer will choose capital, manpower, technology and other production factors to replace the land. This reduces the reliance on land factors and increases output per unit of land area. With the improvement of urbanization, the gradual improvement of electronic information and transportation system will greatly reduce the cost of enterprises, broaden capital channels and expand the flow range of production factors. This not only promotes the factor substitution effect, but also aggravates the spillover of advanced technology and information, and strengthens the effect of inter-city spillover on the green use efficiency of urban land^49^.

The optimization of industrial structure mainly affects GLUE through the "selection effect" and "causative effect". In the process of economic development, the market demand will directly or indirectly interfere with the competitive use of land resources by various economic entities, which increases the production cost of land use. According to the "selection effect", the increase of land use production cost will lead to the exit of low-output and high-pollution enterprises from the market and prevent low-technology-intensive enterprises from entering the market^50^, which leads to the improvement of production efficiency. Regarding the "causative effect", "causative effect" is an important way to improve GLUE by upgrading the industrial structure. The optimization of industrial structure can promote the concentration of capital, technology and talent resources to productive areas with high return on investment, which avoids duplication of construction and promotes the efficient use of land. Therefore, the optimization of industrial structure will promote the improvement of GLUE.

**Figure 2:**
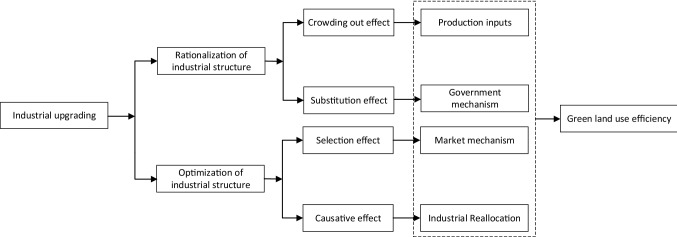
Influence path of Industrial upgrading on GLUE.

Green land use is a long-term and complex process, and the roles of economic and social factors under different productivity are significantly different, which is closely related to the implicit characteristics under different productivity. When the green land use efficiency of is at a low level, such cities show the characteristics of coarse mining of construction land and excessive dependence on land resources. There is huge room for the improvement of GLUE. The optimization of industrial structure plays an important role in the effective use of land. The optimization of industrial structure increases the number of technology-intensive and innovation-driven enterprises, reduces the consumption intensity of resources, and improves the production efficiency. With the continuous improvement of GLUE, simply increasing the proportion of secondary industry and tertiary industry can no longer effectively promote the rational use of land. The rationalization of industrial structure has an increasingly prominent impact on GLUE. On the one hand, the rationalization of industrial structure makes more enterprises with high pollution and high emission forced out of the market, while enterprises with low pollution and high output enter the market, which makes GLUE increase. On the other hand, the rationalization of industrial structure makes more production factors such as capital and talents used. The use of land factors decreases and GLUE increases. With the increase of GLUE level, the promotion effect of industrial structure rationalization on GLUE is enhanced, while the optimization effect of industrial structure on GLUE is weakened. In addition, the resource endowment ability of resource-based cities is different, and the scale of land investment is also different. In cities with abundant resources, it is easy to form the externalities of production factors, which can better match the production factors such as environment, labor, capital and land^51^, in order to form the phenomenon of increasing returns to scale and make the regional economic structure more stable. The effect of economic output is different, which makes the effect of regional land use in resource-based cities different. Therefore, this paper verifies that the green utilization efficiency of land is nonlinear in different areas of resource endowments ability through the threshold effect. In summary, this paper proposes two hypotheses. The influence path is shown in Fig. 2.

### Hypothesis 1

Industrial upgrading can significantly improve the green utilization efficiency of land.

### Hypothesis 2

Industrial upgrading has a nonlinear impact on the efficiency of the green land utilization efficiency.

## Research area, model and method

### Research area

**Figure 3:**
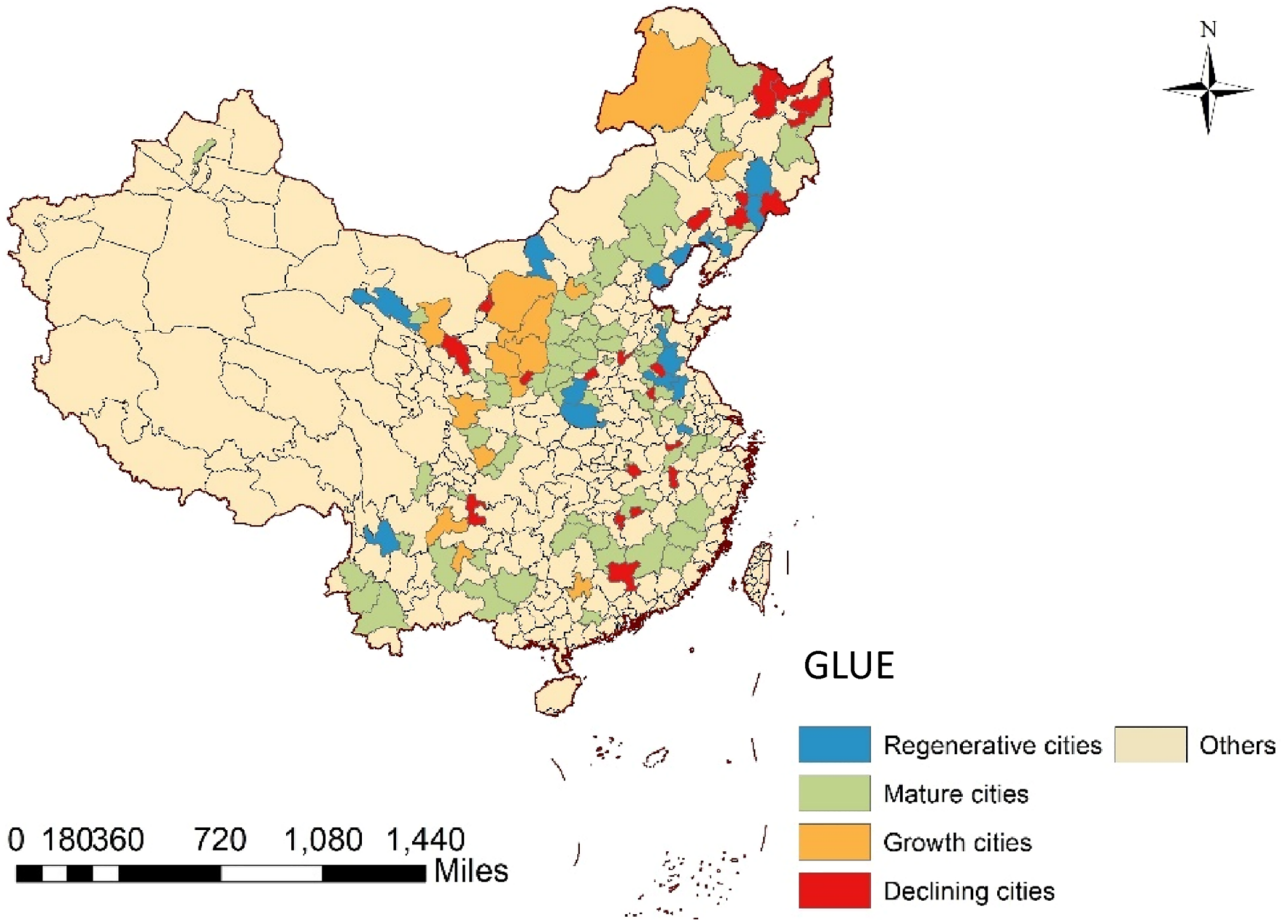
Study area.

As an important guarantee base of resource strategy, the development of resource-based cities is the potential and stable driving force of China’s economic growth. In 2013, the State Council promulgate *Sustainable Development Planning of National Resource-Based Cities*, which identified 262 resource-based cities, including 126 prefecture-level administrative regions, 62 county-level cities, 58 counties (autonomous counties and forest areas) and 16 municipal districts. However, due to the insufficient scale and single industrial structure of county-level cities, counties and administrative regions, it is easy to produce resource crowding-out effect, therefore they are not used as a reference. At the same time, the resource-based cities are mainly divided into four categories, namely, growth type, mature type, decline type and regeneration type. Considering the availability and integrity of data, this paper takes 115 resource-based cities with complete data in prefecture-level administrative regions as samples, which cover 24 provinces in China, including 14 growth cities, 63 mature cities, 23 declining cities and 15 regeneration cities. This article uses ARCGIS 10.2 version to draw the map. The URL link is http://demo.domain.com:6080/arcgis/services. The spatial distribution is shown in Fig. 3.

### Method and model

#### Meta-frontier model

Considering the spatial heterogeneity of green utilization efficiency of land, the common boundary model based on SBM model is adopted in this paper. According to the different technology sets, resource-based cities are divided into four groups. The inter-group frontier model refers to the potential technical level of all DMUs, and the intra-group frontier refers to the actual technical level of DMUs in each group. In this paper, the Meta-frontier model proposed by Battese and Coelli^52^ is used for reference. The specific expressions are as follows:

Assuming that a and b represent input–output vectors respectively, if all sample inputs are included, the common boundary set of output is T^m^

1 $$T^{m} = \left\{ {\left( {a,b} \right):a \ge 0,b \ge 0;a\;{\text{can product b}}} \right\}$$

where the production set corresponding to the above formula may be P^m^

2 $$P^{m} \left( x \right) = \left\{ {b:\left( {a,b} \right) \in T^{m} } \right\}$$

This boundary is the boundary under the common frontier. Therefore, the common distance function under the common technical efficiency is expressed as follows:

3 $$D^{{\text{m}}} \left( {a,b} \right) = \sup_{\varphi } \left\{ {\varphi > 0:\left( {a/\varphi } \right) \in P^{m} b} \right\}$$

Similarly, according to the sustainable development ability of resources and the situation of resource, resource-based cities are divided into four groups (K = 1, 2, 3, 4). Each city refers to the technical set of its group:

4 $$T^{k} = \left\{ {\left( {a_{k} ,b_{k} } \right):a_{k} \ge 0,b_{k} \ge 0;a_{k} {\text{ can product b}}_{k} } \right\}_{{{\text{k}} = 1,2,3,4}}$$

The corresponding set of production possibilities for each group is as follows

5 $$P^{k} \left( {a_{k} } \right) = \left\{ {b:\left( {a_{k} ,b_{k} } \right) \in T^{k} } \right\}_{{{\text{k}} = 1,2,3,4}}$$

This boundary is the boundary under the frontier of group. Therefore, the group distance function under the technical efficiency of the group represents is as follows

6 $$D^{k} \left( {a_{k} ,b_{k} } \right) = \sup_{\varphi } \left\{ {\varphi > 0:\left( {a_{k} /\varphi } \right) \in P^{k} y_{k} } \right\}_{{{\text{k}} = 1,2,3,4}}$$

#### Basic measurement model

Based on the panel data of 115 resource-based cities in China from 2004 to 2018, this paper empirically analyzes the impact of industrial upgrading on GLUE. The following basic measurement models are established:

7 $$GLUE_{it} = \alpha + \beta_{1} RIT_{it} + \beta_{2} OIT_{it} + \delta X_{it} + \mu_{{\text{i}}} + \varepsilon_{it}$$

where i and t represent the region and time respectively ; GLUE, the explained variable, is the green utilization efficiency of land; RIS is the rationalization of industrial structure; β1 represents the influence degree of RIS on green utilization efficiency of land; OIS is the industrial of advanced; β2 means that the influence degree of OIS on the GLUE; Z is the individual effect that does not change with time; X is the control variable; δ represents the coefficient set of the control variable; µ_i_ is the time-invariant individual effect; and ε is the disturbance term. In order to test whether the interpretation variables are related, this paper introduces Hausman test according to^53^ and selects the optimal model^54^.

The above model estimates whether the full sample average will affect the green land use efficiency by industrial upgrading. This paper selects 115 cities in China from 2004 to 2018 as research samples. Due to the significant differences in GLUE between cities, the mean regression ignores the actual situation in the head or tail cities. Quantile regression can obtain conditional distribution, which makes up for the study of extreme values with 'rough tail' or 'spike' samples. Therefore, quantile regression uses various land green use efficiency to model, more comprehensive description of different levels of land green use efficiency affected by industrial transformation. The quantile regression was first proposed by Koenker and Bassett^55^. Quantile regression is less susceptible to variance values, and the results are more robust for models with heteroscedasticity. The quantile regression model is as follows:

8 $$GLUE_{it(\tau )} = \alpha_{\tau } + \beta_{1\tau } RIT_{it} + \beta_{2} OIS_{it} + \delta_{\tau } X_{it} + \mu_{i} + \varepsilon_{it}$$

GLUE_it(τ)_ is the quantile, ατ is the intercept term, and δ_τ_ is the regression coefficient of each control variable. According to the sample distribution, τ is selected as 0.1, 0.25, 0.5, 0.75, and 0.9 to better distinguish the influence of differences.

If the resource endowment between cities is structurally broken, industrial upgrading may have a non-linear impact on the green utilization efficiency of land. The panel threshold model is used to further explore whether there is a non-linear relationship between industries under different resource endowment conditions. In this paper, the panel regression model proposed by Hansen^56^ is used to test the above nonlinear relationship. The panel threshold model is to select the threshold value to divide the model into one or several intervals, and use different regression equations for different intervals. This paper selects the panel threshold model with a single threshold, and the model is as follows:

9 $$GLUE_{{{\text{it}}}} = \alpha + \tau_{1} Z_{it} I(q_{it} \le \gamma_{1} ) + \tau_{2} Z_{it} I(q_{it} > \gamma_{2} ) + \theta X_{it} + \varepsilon_{it}$$

where γ1 is the estimated threshold, qit is the threshold variable, which is the resource endowment, Zit is the endogenous explanatory variable, and I (·) is the exponential function, that is, if the conditions in brackets are met, it is 1, otherwise it is 0, τ1 and τ2 are the estimated coefficients of the dependent variable of the threshold, Xit is the vector of the control variable except the endogenous explanatory variable, α is the intercept term, and ε is the random disturbance term. If there are multiple thresholds, for example two or three thresholds, the double threshold panel model is as follows:

10 $$GLUE_{{{\text{it}}}} = \alpha + \tau_{1} Z_{it} I(q_{it} \le \gamma_{1} ) + \tau_{2} Z_{it} I(\gamma_{1} < q_{it} \le \gamma_{2} ) + \tau_{3} Z_{it} I(q_{it} > \gamma_{2} ) + \theta X_{it} + \varepsilon_{it}$$

In the formula, γ_1_ and γ_2_ are the estimated double thresholds, and the other variables have the same meaning as the above formula.

### Data and variable description

#### Explained variable

Green land use efficiency (GLUE). For the index of green land use, this paper uses the method of Meta-frontier combined with SBM to measure. The key to evaluate the green utilization efficiency of land is to select the appropriate input–output indicators^57^. In the selection of output indicators, it not only needs to consider the social and economic benefits of urban land, but also consider the negative environmental impact in the process of land use, as shown in Table 1.

**Table 1:** Input–output indicators.

Indicator type	Indicator name	Indicator meaning
Input indicators	Urban construction land area	Urban construction land area
	Fixed capital stock	Total investment in fixed assets of the whole society
	Industrial staff	Employees of primary, secondary and tertiary industries in cities
Expected output indicators	Gross domestic product	Urban GDP
Unexpected output indicators	Comprehensive pollution index	Conversion of waste water, waste gas,waste entropy method

Input indicators. Land, capital and labor are selected as input indicators, urban construction land area, fixed capital stock and industrial employees are used to calculate respectively. (1) Urban construction land is the carrier of various activities and the foundation of economic construction^58^. (2) Fixed-asset investment is the sum of the workload and related expenses of social construction and purchase of fixed assets in a certain period of time^45^. (3) Industrial employees are the human resources of social and economic development and the labor base to promote social development^59^.

Expected output Indicators. Expected output indicators reflect the final performance results of urban production and operation activities as well as service activities in a certain period of time, mainly reflecting the market value of all final products (goods and services) produced by production factors. It is a sensitive measure of economic benefits. Therefore, GDP is selected to replace it to reflect social and economic dynamics^60^.

Unexpected output indicators. Industrial waste water discharge, industrial waste gas discharge and industrial waste discharge are collectively referred to as the comprehensive pollution index. The discharge of the comprehensive pollution index is a direct reflection of the negative role of urban economic activities on the environment^61^. In this paper, the entropy method is used to calculate the comprehensive pollution index. The calculation method is as follows:

The weight of waste water, waste gas, waste emissions is determined by using the concept of entropy information.

9 $$M = \left[ \begin{gathered} A_{1} \hfill \\ \vdots \hfill \\ A_{m} \hfill \\ \end{gathered} \right]\left( {\begin{array}{*{20}c} {a_{11} } & \ldots & {a_{1n} } \\ \vdots & \ddots & \vdots \\ {a_{m1} } & \cdots & {a_{mn} } \\ \end{array} } \right)$$

where m is the number of resource-based cities. n is the total number of three wastes

10 $$P_{ij} = \frac{{a_{ij} }}{{\sum\limits_{i = 1}^{m} {a_{ij} } }}$$

The total contribution amount of all schemes to the attribute a_j_ is calculated, where in the total contribution amount of all schemes to the attribute a_j_ can be expressed by E_j_. The calculation method of E_j_ is as follows:

11 $$E_{j} = - K\sum\limits_{i = 1}^{m} {P_{ij} \ln \left( {P_{ij} } \right)}$$

where the constant K =  − 1 / ln ( m ) causes the guarantee 0 =  < E_j_ <  = 1, so that the maximum value of E_j_ is 1, and when the contribution rate of each scheme under a certain attribute tends to be consistent, E_j_ tends to be 1.Especially when the contribution rate is completely equal, the role of the target’s attributes in decision-making can be ignored. D_j_ is the consistency degree of the contribution degree of each scheme under the j attribute, D_j_ = 1 − E_j_. It is the weight proportion in each index, and the weight ratio is multiplied by the value of each index to obtain the index value of the comprehensive pollution index.

#### Explanatory variables

Industrial upgrading (IU). Industrial upgrading is the effect achieved by the coordination between industries and reasonable proportion of various industrial departments, that is, the process of coordination and unification of industrial rationalization and industrial advancement^62^. From a dynamic perspective, the rationalization of industrial structure and optimization of industrial structure jointly affect the industrial structure transformation of the economy, therefore this paper takes the optimization of industrial structure and rationalization of industrial structure as the core explanatory variables.

Rationalization of Industrial structure (RIS). Industrial rationalization refers to the quality of aggregation among industries, which reflects the degree of resource utilization and the coordinated distribution among industries. There are many measurement methods of RIS, such as structural benefit index, deviation degree of industrial structure, standard structure method, etc. According to the differences of economic development, natural resource endowment, labor structure and industrial structure of resource-based cities, the above methods will have certain deviations in the calculation of rationalization of industrial structure, which is not applicable to this paper. Therefore, this paper selects Theil index to describe the rationalization of industrial structure, the calculation formula is as follows:

12 $$RIS{ = }\sum\limits_{{i{ = }1}}^{n} {\left( {\frac{{Y_{i} }}{Y}} \right)} \ln \left( {{{\frac{{Y_{i} }}{{L_{i} }}} \mathord{\left/ {\vphantom {{\frac{{Y_{i} }}{{L_{i} }}} \frac{Y}{L}}} \right. \kern-0pt} \frac{Y}{L}}} \right) = \sum\limits_{i = 1}^{n} {\left( {\frac{{Y_{i} }}{Y}} \right)} \ln \left( {{{\frac{{Y_{i} }}{Y}} \mathord{\left/ {\vphantom {{\frac{{Y_{i} }}{Y}} {\frac{{L_{i} }}{L}}}} \right. \kern-0pt} {\frac{{L_{i} }}{L}}}} \right)$$

where i represents the ith industry of the three industries; n is the total number of industrial sectors.; Y and L represent industrial GDP and labor employment respectively. The calculation of Theil index takes into account the relative importance of various industrial sectors, and avoids the calculation of absolute value and the phenomenon of “equal treatment”, while ignoring the importance of various industry.

Optimization of Industrial Structure (OIS). Optimization of Industrial Structure is mainly the evolution of industrial structure and a measure of industrial upgrading. Its significant characteristics are the decline of the proportion of primary production in total output value, the trend of the significant increase in the proportion of non-agricultural industries. Therefore, this paper selects the proportion of the total output value of the secondary and tertiary industries to the total output value to measure the transformation of industrial structure. If OIS is in an upward state, it proves that the industrial structure is transforming to a positive direction.

#### Control variable

In this paper, several variables that affect the GLUE are used as control variables.

Economic development level (GDPP). This paper uses GDP per capita to represent the economic and wealth status^63^. The green utilization efficiency of land in a country or region changes with the continuous growth of the economy. If the per capita GDP is higher, the city will have more resources and income, which plays a positive role in improving the efficiency of land use. If the economic development is overheated and the land use efficiency of various industries rises, it may lead to negative industries and reduce the green utilization efficiency of land. In order to be more persuasive and comparable, this paper uses 2004 as the base period, expressed at constant price.

Foreign direct investment (FDI). The degree of foreign direct investment is measured by the degree of trade openness^43^, which is expressed by the actual use of foreign capital as a percentage of GDP in that year. Due to the industrial form and industrial development level of different countries exist difference, according to the theory of factor endowment, when opening up foreign trade, it may not only lead to the direction and efficiency of land use exist differences, but also change the promotion of land use worldwide. Even if there is no policy of industrial structure, the implementation of China’s opening up policy may also promote the change the green utilization efficiency of land.

Technical efficiency change (TEC). Scientific and technological progress is the first productive force, obviously, major changes and breakthroughs should be achieved through technological innovation, green land use is the same. According to Wu and Liu^64^, this paper uses the proportion of the number of patents to the GDP to represent technical progress, in which the number of patents is invention patents authorization * 4 + utility model * 2 + design * 1.

Degree of government intervention (DGI). Government regulation and control policies have a great impact on regional land use^65^. The bottleneck of land use efficiency can only be broken if the government provide sufficient supports for improving the land use efficiency. The main form of government regulation is the government expenditure, so the degree of government regulation is expressed by the ratio of local fiscal expenditure to GDP.

Population growth rate (PGR). Population provides labor productivity for social development^66^, which is one of the three elements of development. At the same time, the emergence of population agglomeration and other phenomena drives the change of land resource use, and the increase of population density will directly affect the efficiency of land use.

The data in this article come from *China Statistical Yearbook*, *China Urban Statistical Yearbook*, *China Environmental Statistical Yearbook* and the State Intellectual Property Office. The article data were smoothed and logarithmically processed. The statistical table of each variables is described as shown in Table 2.

**Table 2:** Descriptive statistics of variables.

	N	Mean	SD	Min	Max
GLUE	1725	0 0.961	0.222	0.008	2.414
RIS	1725	5.131	1.147	0.002	7.450
OIS	1725	5.131	0.106	1.612	2.301
PRG	1725	5.305	4.846	− 16.270	39.180
DGI	1725	7.218	0.596	4.588	11.155
FDI	1725	4.236	1.457	− 4.134	7.672
TEC	1725	1.708	1.207	− 1.541	8.283
GDPP	1725	9.452	0.590	6.945	11.435

### Ethics approval and consent to participate 

This study involves the macro data of human economy and society. All the data are from the official statistical yearbook. The data collection process is in line with the ethical and moral standards. The authors guarantee that the process, content and conclusion of this study do not violate the theory and moral principles.

## Empirical results

### Temporal and spatial distribution characteristics

**Figure 4:**
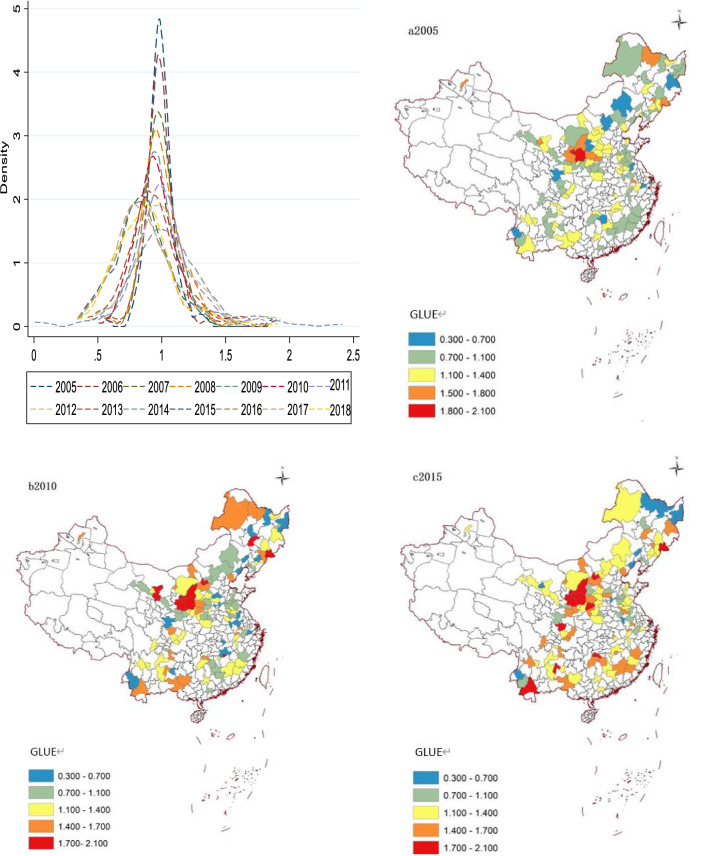
Spatial and temporal characteristics of GLUE.

Figure 4 shows the density distribution and spatial distribution characteristics of GLUE in resource-based cities in 2005, 2010 and 2015. Map drawing software is consistent with the above figure. Due to the influence of development level, resource saturation and richness, different regions show a characteristic of heterogeneity. In terms of spatial characteristics, 115 cities show the characteristics from north to south, from high to low. Specifically, the northern region is rich in resources with comparative advantages, and it has sufficient capital investment for the innovation of green land utilization technology to form the “gospel” effect of resources. However, in the southern region, due to the high population density, cities are relatively dense and available land resources are relatively scarce, it is more difficult to improve the GLUE in the southern region. On the contrary, the northern region has external advantages, and the rational development and use of land is also more adequate. With the advanced of time, the gap of GLUE in various provinces is further expanded. The concentration of cities is reduced, which makes GLUE appear “polarization”. Although inefficiency cities are gradually improving, there is still a certain gap among the more efficiency cities.

### Empirical results of panel data model

This paper uses a variety of regression methods to analyze the basic regression relationship between variables, including mixed regression model, fixed effect model (FE), random effect model (RE). In order to accurately obtain the quantitative relationship between variables accurately, F test, LM test, Year test and Hausman test are used to select the model. Since the structural upgrading includes two dimensions, this paper takes both RIS and OIS as the core explanatory variables. For obtaining more convincing conclusions, this paper takes these two dimensions and their interaction items as the core explanatory variables to explore their effects on the explained variables.

In Table 3, columns (1), (2), (3), (4) and (5) represent the estimated results of mixed OLS model, random effect model, year fixed effect model, city fixed effect model and year and city dual fixed effect model with industrial structure upgrading and industrial structure rationalization as the core explanatory variables. According to the results of F test and LM test, the mixed OLS model should not be used. The Year test results show that the model should not only consider the year effect, but also consider the city effect. Therefore, this paper should use the year and city dual fixed effects model or random effect model for empirical analysis. The Hausman test results show that the dual fixed effects model is more suitable than the random effect model. Therefore, the dual fixed effect model is selected to interpret the estimated results. Columns (6), (7) and (8) are the estimation results of the dual fixed effect model with the rationalization of industrial structure as the single core explanatory variable, the upgrading of industrial structure as the single core explanatory variable and the interaction term of industrial structure, respectively.

**Table 3:** Basic regression results.

	(1)	(2)	(3)	(4)	(5)	(6)	(7)	(8)
RIS	0.024***	0.016***	0.017**	0.013**	0.012**	0.009		
	(4.77)	(2.86)	(2.25)	(2.00)	(2.01)	(1.58)		
OIS	0.273***	0.130**	0.155**	0.219***	0.147**		0.125**	
	(4.76)	(2.55)	(1.99)	(2.61)	(2.44)		(2.10)	
RIS*OIS								0.005*
								(1.85)
PGR	0.003***	0.003***	0.003***	0.003**	0.003**	0.002**	0.003***	0.002**
	(2.61)	(2.64)	(2.66)	(2.54)	(2.48)	(2.39)	(2.80)	(2.37)
DGI	− 0.024**	− 0.029***	− 0.020***	− 0.019***	− 0.030***	− 0.030***	− 0.029***	− 0.031***
	(− 2.51)	(− 3.24)	(− 3.01)	(− 3.25)	(− 3.38)	(− 3.36)	(− 3.26)	(− 3.39)
FDI	− 0.010***	0.007**	0.007**	0.010**	0.010**	0.009*	0.009***	0.009***
	(− 2.85)	(1.97)	(2.33)	(2.34)	(2.70)	(2.58)	(2.61)	(2.60)
TEC	− 0.054***	− 0.051***	− 0.052***	− 0.051***	− 0.051***	− 0.051***	− 0.051***	− 0.051***
	(− 11.84)	(− 12.52)	(− 12.22)	(− 12.25)	(− 12.22)	(− 12.21)	(− 12.39)	(− 12.19)
GDPP	0.054***	0.131***	0.142***	0.109***	0.154***	0.158***	0.156***	0.157***
	(5.09)	(10.57)	(10.79)	(11.05)	(11.39)	(11.66)	(11.50)	(11.58)
_cons	0.028	− 0.386**	− 0.447	− 0.522**	− 0.626***	− 0.321**	− 0.536***	− 0.320**
	(0.17)	(− 2.16)	(− 1.58)	(− 2.55)	(− 3.25)	(− 2.19)	(− 2.86)	(− 2.19)
R^2^	0.17	0.22	0.22	0.22	0.22	0.22	0.22	0.22
LM		3349.13***						
F		30.25***	15.58***	15.77***	16.63***	19.96***	20.00***	20.25***
Year test			2.55***					
Hausman test			26.21***	30.45***	27.51***	31.35***	19.76***	20.83***
Year fixed effect	No	No	Yes	No	Yes	Yes	Yes	Yes
City fixed effect	No	No	No	Yes	Yes	Yes	Yes	Yes
N	1725	1725	1725	1725	1725	1725	1725	1725

*p < 0.1, **p < 0.05, ***p < 0.01; the bracket represents the t value.

According to the column (5) of Table 3, the results of the regression coefficients of RIS and OIS are significantly positive, and when the RIS increases by 1%, the GLUE increases by 0.012%. When OIS increased by 1%, GLUE increased by 0.147%, indicating that both the OIS and RIS had a positive impact on the GLUE. This also confirms hypothesis 1. It is worth noting that although the coefficient of RIS is positive, the coefficient of this indicator is much smaller than that of OIS. It can be seen that the impact of OIS on GLUE is much greater than that of RIS; It is found that the coefficient and significance of RIS and OIS have little change, and both have a positive impact on GLUE. Other control variables also provide corresponding information. The regression coefficients of PGR, FDI and GDPP are positive. The increase in population growth rate can provide human factors for production, and the opening of regions and the improvement of economic development level can bring sufficient capital to social production. Driven by the tightening cost of land and the growth of profits, enterprises will adjust the ratio of factor input, and the substitution of human and capital for land can increase the degree of dependence on land, thereby improving the GLUE. The regression coefficients of DGI and TEC are negative, indicating that the government's control and technological progress are still in the exploratory stage and cannot improved GLUE.

### Robustness test

#### Variables and model substitution

Firstly, GLUE is calculated by Metafrontier− Malmquist index, and it takes land, capital and labor as input indicators, and adopts economic benefits and negative environmental benefits as output indicators. The total factor productivity in the frontier is regarded as the alternative index of green land use efficiency. Secondly, the ratio of science and technology expenditure to total financial expenditure in government expenditure is regarded as a substitute indicator for OIS in the stability test^67^. Finally, in order to solve the heteroscedasticity problem, the model is replaced with the model under the clustering robust standard error. Model (9), model (10), and model (11) in Table 4 represent mixed OLS, the estimated results of the year and city dual fixed effects and random effects models respectively. Compared with Table 4, the explanatory variables of each model are completely consistent in symbols in addition to the differences in the correlation coefficient and significance level. The empirical results are consistent with the above results, indicating that the above estimations are robust and reliable.

**Table 4:** Robustness test results.

	(9)	(10)	(11)
RIS	0.014** (2.14)	0.044** (5.45)	0.028*** (3.78)
OIS	0.039** (2.02)	0.016* (1.88)	0.036 (1.39)
PGR	0.006 (1.16)	0.002 (1.21)	0.002 (1.55)
DGI	− 0.035 (− 1.54)	− 0.011 (− 0.15)	− 0.008 (− 0.77)
FDI	0.008* (− 1.93)	0.008** (2.08)	0.004* (1.62)
TEC	− 0.011** (− 2.56)	− 0.022** (− 2.16)	− 0.039*** (− 3.46)
GDPP	0.074*** (6.22)	0.015* (1.91)	0.100** (2.44)
_cons	− 0.182*** (− 3.32)	− 0.258*** (− 2.59)	− 0.206** (− 1.99)
R^2^	0.39	0.32	0.23
LM			1028.29***
F test		98.63***	58.55***
Year fixed effect	No	Yes	No
City fixed effect	No	Yes	No
Hausman test		19.98***	19.98***
N	1725	1725	1725

*p < 0.1, **p < 0.05, ***p < 0.01; the bracket represents the t value.

#### Classification discussion

*Sustainable Development Planning of Resource—Based Cities in China* points out that the resource-based cities in China are divided into four types according to the sustainable development capacity and resource situation, which are regenerative cities, growth cities, mature cities and declining cities. Therefore, in order to investigate the regional heterogeneity effect of industrial upgrading on the green utilization efficiency of land, this paper performs a classification analysis for the resource-based cities. In Table 5, Cluster1, Cluster2, Cluster3 and Cluster4 represent regenerative cities, growth cities, mature cities and declining cities respectively. Industrial structure upgrading has obvious regional heterogeneity on the green utilization efficiency of land. The rationalization of industrial structure has a positive coefficient in regenerative cities, growth cities and declining cities, which shows that rationalization of industrial structure can play a more significant role in cities with resources still in the stage of development. Regenerative cities and growth cities are more likely to provide intermediate inputs needed for rationalized production technology, and knowledge spillover and technology spillover between different industries are more significant. The resource endowment of mature and declining cities is weak, reaching the critical value of overuse. Obviously, these cities may be difficult to profit from different industries because of the competition for the same factor of production and the increase of factor costs, thus the rationalization of industrial structure has a weak or even negative effect on efficiency. From the perspective of labor force, regenerative cities and growth cities have more dynamic markets. Labor force can diversify the flow among industries, promote employment and population mobility, and make inter-industry allocation more reasonable, thus promoting the efficiency. In terms of the optimization of industrial structure, corresponding to the rationalization of industrial structure, the coefficients of regenerative cities, growth cities and declining cities are positive. Due to excessive reclamation of resources in declining cities, the market with weak resource shrinkage and endowment can easily form a monopoly market, which can easily lead to market rigidity and weak self-regulation ability. However, through the upgrading of structural classification and changing the proportion of industrial structure changes, it will play a significant role in promoting the land efficiency of declining cities. For mature cities, the utilization of resources has reached a certain level, the degree of matching between industries is higher. The phenomenon of excessive external competition, shortage of public resources and excessive industrial competition weakens the positive external effect, thus inhibiting land use efficiency.

**Table 5:** Grouped regression results.

	Cluster1	Cluster2	Cluster3	Cluster4
RIS	0.029** (1.99)	0.025*** (2.62)	− 0.031 (− 1.50)	0.012 (1.51)
OIS	0.114 (0.53)	0.106 (1.34)	− 0.528** (− 2.36)	0.486*** (4.84)
PGR	0.007*** (2.98)	0.010 (0.62)	− 0.001 (− 0.50)	0.005*** (2.63)
DGI	− 0.034 (− 1.36)	− 0.003 (− 0.26)	− 0.036 (− 1.47)	− 0.066*** (− 3.70)
FDI	0.011 (1.26)	0.007 (1.32)	0.034*** (2.85)	0.008 (1.46)
TEC	− 0.051*** (− 5.69)	− 0.057*** (− 9.68)	− 0.047*** (− 3.51)	− 0.043*** (− 5.50)
GDPP	0.001 (0.04)	0.109*** (5.67)	0.326*** (9.06)	0.204*** (6.98)
_cons	0.789 (1.39)	− 0.349 (− 1.36)	− 0.410 (− 0.66)	− 1.625*** (− 4.13)
*N*	225	945	210	345
F	10.48***	13.53***	18.47***	27.71***
LM	179.51***	1180.39***	151.45***	847.54***
R^2^	0.29	0.19	0.37	0.44
Hausman	3.50	74.85***	43.03***	4.42

*p < 0.1, **p < 0.05, ***p < 0.01; the bracket represents the t value.

#### Endogenous problems

The optimization of industrial structure and the rationalization of industrial structure, as explanatory variables, may exist endogenous. In order to solve the regression error caused by endogenous problems in the model, this paper uses the instrumental variable (IV) method to deeply analyze the causal relationship between industrial upgrading and GLUE, as shown in Table 6. In the selection of instrumental variables, this paper selected the lag term of OIS, the lag term of RIS and the interaction term of land slope and year (LPY) as instrumental variables. With regard to the instrumental variable of OIS and RIS lag term, industrial upgrading has a certain inertia effect, the previous industrial transformation will affect the current effect, so it meets the requirements related to endogenous variables. The current GLUE cannot affect the industrial transformation in the previous period, avoiding the possibility of reverse causality. As for the instrumental variable of the interaction between land slope and year, land slope will have an impact on social production activities. The steeper the land, the more difficult it is for industrial production, which is conducive to the development of tourism and service industry, thus affecting the layout of industrial structure. Therefore, the tool variable meets the correlation requirement. Land slope is a geographical factor, which will not directly affect GLUE. Therefore, the tool variable meets the exogeneity requirement.

**Table 6:** Estimation results of instrumental variable method.

Instrumental variables	L.OIS	L.RIS	LPY	
	(12)	(13)	(14)	(15)
OIS	0.293*** (3.77)	0.318*** (5.20)	0.094** (2.50)	0.112** (2.55)
RIS	0.026*** (4.87)	0.033*** (4.78)	0.077** (2.48)	0.085** (2.28)
PGR	0.003** (2.55)	0.002** (2.00)	0.001 (1.22)	0.001 (1.00)
DGI	− 0.024** (− 2.40)	− 0.022** (− 2.17)	0.007 (1.48)	0.007 (1.33)
FDI	− 0.010*** (− 2.79)	− 0.010** (− 2.72)	− 0.019** (− 2.00)	− 0.025*** (− 2.65)
TEC	− 0.060*** (− 12.23)	− 0.061*** (− 12.35)	− 0.037*** (− 10.95)	− 0.043*** (− 11.22)
GDPP	0.057*** (4.68)	0.059*** (5.09)	0.048* (1.70)	0.035 (1.56)
_cons	− 0.031 (− 0.16)	− 0.162 (− 0.85)	0.132 (1.12)	0.127 (1.17)
*N*	1610	1610	1725	1725
R^2	0.19	0.19	0.24	0.24
Control	Yes	Yes	Yes	Yes
F test	51.26***	52.56***	33.25***	34.42***
Root MSE	0.207	0.207	0.117	0.117
First stage IV	0.830***	0.753***	0.712***	0.701***

*p < 0.1, **p < 0.05, ***p < 0.01; the bracket represents the t value.

According to Table 6, the effect of the core explanatory variables on explained variables is still positive. According to the regression results of the first stage model, the lag terms of OIS and RIS have certain explanatory power for OIS and RIS respectively. F test shows that IV is significant, when using industrial variables for OIS, as shown in columns (12) and (14) of Table 6, when OIS increases 1%, GLUE increases about 0.293% and 0.094%, respectively. When RIS increased by 1%, GLUE increased by about 0.026% and 0.033%, respectively. When the instrumental variables are used for RIS, as shown in column (13) and (15) of Table 6, when OIS increases by 1%, GLUE increases by about 0.318% and 0.112% respectively. When RIS increases by 1%, GLUE increases by about 0.033% and 0.085%, respectively. The general trend remains unchanged, indicating that after eliminating the endogeneity of OIS and RIS, GLUE is still increased.

### Quantile regression analysis

Since the basic regression model can only reveal the average impact of one economic phenomenon on another new economic phenomenon, it ignores the possible "thick tail" or "peak" characteristics of economic variables, and conceals the importance of extreme values of economic variables. The quantile regression makes up for the shortcomings of the basic regression to obey the normal distribution, can give the estimated values of the explanatory variables corresponding to different quantiles, and is less sensitive to data outliers and can overcome extreme values. According to hypothesis 2, considering the heterogeneity of the distribution of GLUE, this paper further performs quantile regression on the intensity of GLUE. The results of the models (16), (17), (18), (19) and (20) in Table 7 are shown.

**Table 7:** Quantile regression results.

	(16)	(17)	(18)	(19)	(20)
OIS	0.361*** (5.26)	0.307*** (4.49)	0.248*** (4.14)	0.014** (2.21)	0.052 (0.29)
RIS	0.007 (1.57)	0.018*** (3.65)	0.018*** (3.84)	0.016 (0.15)	0.039*** (3.15)
PGR	0.006*** (5.91)	0.003*** (3.07)	0.002** (2.37)	0.001 (0.84)	− 0.001 (− 0.44)
DGI	− 0.078*** (− 7.40)	− 0.064*** (− 7.39)	− 0.038*** (− 4.59)	− 0.001 (− 0.09)	0.029 (1.47)
FDI	0.008* (1.91)	0.001 (0.02)	− 0.009** (− 2.35)	− 0.016*** (− 3.28)	− 0.032*** (− 3.06)
TEC	− 0.063*** (− 10.65)	− 0.049*** (− 12.28)	− 0.039*** (− 9.77)	− 0.040 (− 5.91)	− 0.040*** (− 2.85)
GDPP	0.012 (0.89)	0.019* (1.70)	0.038*** (3.39)	0.080*** (6.02)	0.132*** (5.58)
_cons	0.428* (2.07)	0.426*** (3.20)	0.314** (2.23)	0.322 (1.43)	− 0.373 (− 0.90)

*p < 0.1, **p < 0.05, ***p < 0.01; the bracket represents the t value.

Firstly, the coefficients of OIS and RIS on GLUE are all positive at the 0.1, 0.25, 0.5, 0.75, and 0.9 quantiles, indicating that within the research sample of this study, the impact of industrial upgrading on GLUE is always positive. This is because, with the continuous increase in GLUE, the transformation of the "targeted" industrial structure has been realized, and the effect of industrial upgrading has been brought into full play. Secondly, when it is less than the 0.75th percentile, the influence coefficient and significance of OIS on industrial GLUE both decrease. For every 1% increase in OIS, GLUE increases by 0.361%, 0.307%, 0.248% and 0.014% respectively. RIS shows a trend of increasing volatility. For every 1% increase in RIS, GLUE increases by 0.007%, 0.018%, 0.018%, and 0.016%, respectively. When it is greater than the 0.75th percentile, the change of OIS is relatively smooth, and the significance is significantly reduced. The influence coefficient and significance of RIS both increased rapidly. Therefore, on the whole, OIS shows a trend of decreasing volatility, and RIS shows a trend of steady increase. Under all quantiles, with the increase of quantiles, the role of industrial upgrading gradually decreases, and the role of RIS gradually increases. This shows that the internal improvement of the region is more important than the external growth. The development of the region is a process of innovation first and then rationalization. The improvement of green land utilization efficiency is not only dependent on the development of a single industry, but coordinated development is the only way to make the land green. Use the key from "high speed" to "high quality".

**Figure 5:**
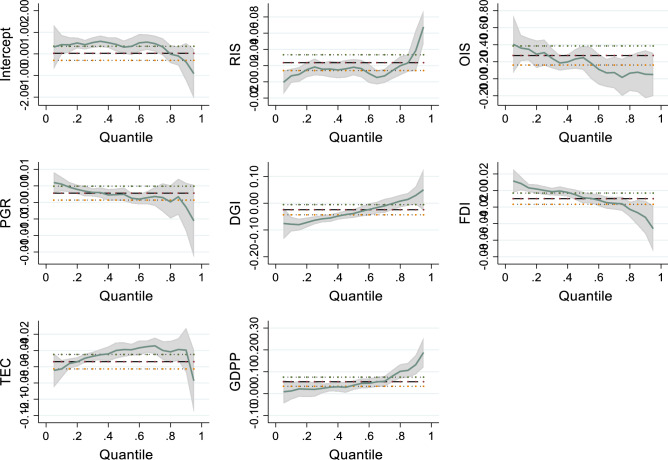
Quantile regression coefficient trend graph.

Figure 5 further shows how the quantile regression coefficients change with quantiles, and the figure below shows the quantile regression at the 95% confidence level.

### Nonlinear threshold effect

The above research proves that there is a positive correlation between industrial upgrading and the green utilization efficiency of land, and further discusses whether there is a nonlinear relationship between industrial upgrading and the green utilization efficiency of land. This paper uses the resource endowment variable (RE) as the threshold variable. The overall concept of resource endowment refers to the total amount of natural resources owned by the economy at a certain time point^68^. In this paper, the employees of the mining industry at the end of the year are used to measure the resource endowment. The mining industry mainly includes coal, oil, natural gas, metal and non-metallic mining and dressing industry, timber harvesting and the production and supply of tap water, which are directly related to natural resources. It can comprehensively and accurately measure the dependence of the economy on natural resources and accurately analyze the role of industrial transformation. When GLUE is used as the explanatory variable, the resource endowment of 115 resource-based cities have no threshold value, one threshold value and two threshold values are analyzed respectively. By drawing on Hansen^56^ “bootstrap method” (bootstrap) to obtain the corresponding threshold value and P value via repeating 1000 times sampling, it can identify whether the model has a threshold value and several threshold values.

In order to fully explain the impact of industrial upgrading on green utilization efficiency of land under the effect of resource endowment, this paper takes the interaction terms of RIS and OIS, RIS and OIS as a single endogenous variable respectively. As shown in Table 8, when RIS is used as a single endogenous explanatory variable, the threshold is significant at the 1% confidence level of in the (21) column. Similarly, the column (22) and (23) show the result when OIS, the interaction terms of RIS and OIS are used as a single endogenous explanatory variable respectively.

**Table 8:** Threshold effect test results.

Threshold	(21)		(22)		(23)	
	F value	P value	F value	P value	F value	P value
One threshold	62.96	0.0020***	75.70	0.0010***	67.69	0.0040***
Double threshold	11.60	0.8370	17.54	0.5690	10.96	0.8560
Three threshold	8.30	0.9590	11.76	0.9220	8.45	0.9530

*p < 0.1, **p < 0.05, ***p < 0.01; the bracket represents the t value.

With resource endowmentas the threshold variable, it can be found that one threshold is significant at the 1% significance level, while double threshold and three threshold are not significant. Using bootstrap to calculate the 95% confidence interval of the threshold, Table 9 indicates that when RIS, OIS, and interaction terms of RIS and OIS are regressed with the threshold variable, the regression values of the three models are the same, which are 37,082, 68,600 and 106,700, however, there are certain differences in the 95% confidence interval.

**Table 9:** Threshold estimation results.

Threshold	Threshold value	(24)	(25)	(26)
		95% critical level	95% critical level	95% critical level
One threshold	37,082.00	(36,175.50 37,500.00)	(36,451.00 37,500.00)	(36,451.00 37,500.00)
Double threshold	37,082.00	(36,175.50 37,500.00)	(36,175.50 37,500.00)	(36,175.50 37,500.00)
	68,600.00	(65,860.50 69,800.00)	(66,410.50 69,800.00)	(65,860.50 69,800.00)
Three threshold	106,700.00	(96,100.00 108,264.00)	(100,635.50 108,264.00)	(96,850.00 108,264.00)

**Figure 6:**
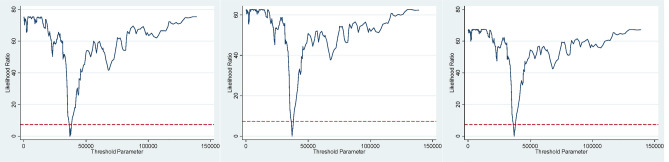
Confidence interval construction.

According to the panel regression results, this paper obtains the likelihood ratio function diagram of the three threshold estimates under the 95% confidence interval, when RIS, OIS and interaction terms of RIS and OIS are used as endogenous variables in resource-based cities. As shown in Fig. 6, the true threshold value corresponding to the lowest point of LR statistics is significantly lower than the critical value of 7.3523, which can be seen that the three models are nonlinear in the regression and the single threshold is real and effective. Therefore, the three regression models are all using one threshold model.

When the threshold values of the three models are obtained, it can obtain the regression results of the single endogenous variable at the same time. As shown in Table 10, columns (27), (28) and (29) are the regression results taking RIS, OIS, and the interaction term between RIS and OIS as the single endogenous variable. It can be seen that different threshold intervals have different effects on the efficiency of land, especially when the endogenous explanatory variable is RIS and the interaction term between RIS and OIS, the coefficients in the model regression results are quite different. On the one hand, when the resource endowment is weak (RE ≤ 37,082), the coefficient of RIS is 0.0036,the coefficient of OIS is 0.128,the coefficient of interaction term between RIS and OIS is 0.0027. On the other hand, when the resource endowment is strong (RE > 37,082), the coefficient of RIS is 0.0306, the coefficient of OIS is 0.1960, the coefficient of interaction term between RIS and OIS is 0.0155. Meanwhile, the results of each control variables are significant, and the influence direction of the explanatory variables is consistent with the results of the double endogenous variables except the regression coefficient and significance. Therefore, it further proves that the beneficial impact of industrial structure on the green utilization efficiency of land gradually rise with the increasing of resource endowment ability. When the threshold value exceeds 37,082, the effect of industrial upgrading on GLUE is more obvious.

**Table 10:** Parameter estimation results of panel regression model.

Variable	(27)	(28)	(29)
PGR	0.0022** (2.23)	0.0024** (2.56)	0.0021** (2.19)
DGI	− 0.0261*** (− 2.94)	− 0.0242*** (− 2.74)	− 0.0263*** (− 2.96)
FDI	0.0083** (2.40)	0.0088** (2.55)	0.0086** (2.47)
TEC	− 0.0521*** (− 12.73)	− 0.0527*** (− 12.96)	− 0.0518*** (− 12.69)
GDPP	0.1493*** (11.21)	0.01472*** (11.06)	0.1482*** (11.13)
RIS (RE ≦ 37,082)	0.0037** (0.63)		
RIS (RE > 37,082)	0.0306*** (4.81)		
OIS (RE ≦ 37,082)		0.1280** (2.20)	
OIS (RE > 37,082)		0.1958*** (3.33)	
RIS*OIS (RE ≦ 37,082)			0.0027 (0.99)
RIS*OIS (RE > 37,082)			0.0155*** (5.18)
_cons	− 0.2787	− 0.5392	− 0.2810
R^2^	0.2533	0.2533	0.2495
F value	21.50***	21.50***	21.14***
N	1725	1725	1725

*p < 0.1, **p < 0.05, ***p < 0.01; the bracket represents the t value.

### Result discussion

Firstly, through the fixed effect model, it verifies that the optimization and rationalization of industrial structure play a positive role in improving the green utilization efficiency of land. Industrial upgrading can improve GLUE by influencing government management, producer, consumer behavior and specialization. First, government policy-making and implementation should be based on changes in industrial structure. Policy will have a role in promoting the industrial structure level, which is conducive to improving the goal of GLUE. Second, industrial upgrading leads to changes in the supply and demand of resources. On the one hand, it causes the redistribution of resources among industries. This has accelerated the elimination of backward production capacity, process equipment and products such as electricity, coke, calcium carbide, steel, non-ferrous metals, building materials and textiles. On the other hand, it has vigorously promoted the development of new energy, new medicine, new materials, sensor networks and other emerging industries. In this process, there will be a variety of industrial location trends. Changes in social demand and supply affect the production of the industrial chain. According to the theory of “consumer rule”, consumers maximize economic benefits through “money vote”. So as to realize the pursuit of emerging technologies and high-tech products, eliminate mature and backward industries. Therefore, the phenomenon of new industries expelling mature industries will occur. Industries transfer in space, mature industries migrate out of the urban center. This leads to changes in the proportion of factory buildings and supporting land, the proportion of production service land construction, and the proportion of green area. The proportion of productive land and high-tech industrial land will be increased, so that the land use structure will be optimized. Finally, industrial upgrading will lead to the development of productive forces, which will lead to the transfer of resources from inefficient production sectors to highly efficient production sectors. The development of specialized production will promote the progress of technology and the formation of “aggregation effect”. In the process of resource flow, new functions, new industries, new space requirements and land mix have been generated, which have contributed to changes in land use performance. Capital flow has changed the transformation of leading industries and the ability of enterprises to earn profits and pay land rents, forcing urban land restructuring. Finance, insurance, firms and other business and service industries will occupy the central part of the city. Industries with high pollution, high consumption and low efficiency will be far away from the urban center.

For example, Tangshan City has a unique geological structure, abundant coal resources and rich history of coal mining. In 2005, the Ministry of Land and Resources approved the establishment of Tangshan Kailuan Mine Park, the scenic area is connected by the mine-owned railway, realizing the upgrading of industry to tourism. Moreover, with the continuous improvement of the industrial chain and the continuous attention to the sustainable development of green land use, China has formulated a series of policies on upgrading and optimizing the production and green utilization of land. The State Council successively promulgates the policies of *Guidance on Accelerating the Development of Producer Services to Promote the Adjustment and Upgrading of Industry and Structure*, *Opinions on Supporting Industrial Transformation and Upgrading of Old Industrial Cities and Resource-based Cities*, *General Outline of National Land Use Planning*. In order to realize the upgrading of the industrial chain and the reuse of land, the government also puts forward the “13th Five-Year Plan” with innovation, harmony, ecology, openness and sharing^69^. These policies aim to promote the utilization rate of natural resources and realize the optimization within the industrial structure. The improvement of the distribution of natural resources has realized the high-end upgrading of the industrial chain and the reuse of land. Therefore, the government should pay more attention to the coordination of land use planning and economic development. Through the establishment of science and technology parks and high-tech industrial parks, the government should optimize the internal structure of the industry, attract more high-tech labor force and form a virtuous circle within the industry, so that it can play a “follow-up motive power” to improve the efficiency of urban land use.

Secondly, green land use is a long-term and tortuous process. The optimization of industrial structure is the industrial reallocation of production resources to a higher gradient and the upgrading of socially dominant industries, which is mainly manifested in the evolution of the proportional relationship of various industries and the improvement of production efficiency. It completes industrial replacement by transforming traditional industries, eliminating backward industries, and cultivating emerging industries, so as to achieve industrial optimization and upgrading. The resulting new industrial spatial pattern will generate new demand for land use, which will lead to the continuous upgrading of land use structures such as changes in land quantity, functional conversion, and spatial reconstruction. The rationalization of industrial structure is the coupling degree of input and output structure of reaction factors, the dynamic process of coordination ability between reaction industries and the continuous improvement of correlation level. For the stage with low level of green land utilization efficiency, cities will easily reach a consensus on the development of resource-based industries, the industrial structure will form a path-dependent progression. The development of resource-based areas is prone to path dependence. Because the pollution emission intensity of the tertiary industry is lower than that of the secondary industry. As a result, cities with high resource abundance have higher pollution intensity and more prominent environmental problems. Based on the "pressure effect", the government has raised the standards of environmental regulation to a certain extent and accelerated the transformation of the industry to the tertiary industry. The role of the optimization of industrial structure industrial structure is more prominent.

Finally, through the threshold effect model, it verifies that the green land utilization efficiency is nonlinear in different areas of resource endowment. When the threshold variable exceeds the threshold value, industrial upgrading tends to be beneficial to GLUE. For different regions with different resource richness, the effect of the implementation of industrial structure policy varies greatly in different spatial scales in China. In the case of different sustainable development of resources, the focus of development is different, and the effect of government policy implementation is also different, so that the resource matching methods are different. On the one hand, resource endowment is the embodiment of resource abundance. In areas with weak resource endowment, the exploitation of natural resources has reached the upper limit, the horizontal adjustment of rationalization can no longer have a significant effect on efficiency. It can only rely on the advanced vertical upgrade, change the structural system, and weave the matrix network of production structure. In areas with strong resource endowment, natural conditions are superior, and appropriate horizontal adjustment can achieve “efficient reporting”. Land resources can be flexibly allocated, which is easy to produce strong external effects and form economies of scale, so as to achieve the trend of increasing economies of scale, drive the rational use of resources and boost the improvement of land use efficiency. On the other hand, resource endowment directly determines the dependence of the industry on natural resources. The stronger the dependence on resources, the greater the intensity of sewage discharge, leading to prominent ecological problems, it will lead to a stronger influence on green land use. In summary, if cities are divided into several regions according to the resource endowment ability, cities with high resource endowment will have a greater impact on the GLUE by upgrading their industrial structure, and the resource “Gospel” effect is stronger. In areas with different natural endowments, relevant policies should be adopted according to the emphasis of urban development and local conditions to achieve optimal allocation of resources, so as to promote GLUE.

## Conclusions and policy recommendations

### Conclusions

Based on the panel data of 115 resource-based cities in China from 2004 to 2018, this paper uses the basic regression and quantile regression model to empirically study the effectiveness of industrial upgrading on the green land utilization efficiency. It finds that in resource-based cities, OIS and RIS have a positive pulling effect on GLUE. Technological progress and government regulation have a negative effect on green land use, while economic development, regional openness and population growth rate have pulling effects on the GLUE. When continuing to conduct regional analysis of GLUE, the results of the four different characteristic regions are various. However, the utility of industrial upgrading is significantly positive on the whole. When discussing quantiles, the effects of OIS and RIS on GLUE are positive at the 10th, 25th, 50th, 75th and 90th quantiles. On the whole, OIS shows a trend of decreasing volatility, and RIS shows a trend of steady increase. Under all quantiles, with the increase of quantiles, the role of industrial upgrading gradually decreases, and the role of industrial structure rationalization gradually increases. In the threshold effect test, the impact of industrial structure upgrading on green utilization efficiency of land will enhance with the increase of resource endowment. When crossing the threshold value, the green utilization efficiency of land eventually develops in an appropriate direction. These findings provide enlightening insights into the GLUE in industrial upgrading.

### Policy recommendations

According to theoretical analysis and empirical research, this paper puts forward the following policy recommendations: (1) Industrial upgrading can effectively promote the improvement of land green utilization efficiency. The government should increase policy support and management efforts for industrial upgrading, establish a sound industrial chain structure, and make it play the "follow-up power" of improving GLUE. At the same time, the government should adjust the mode of land use, encourage the mixed mode of land use in which multiple industries coexist, advocate the unified layout of manufacturing and service industries with strong technical linkages, and speed up the association and interaction between urban industries, so as to realize technology and knowledge spillover between industries. (2) Based on quantile level and threshold effect, for cities in different stages of sustainable development, the government should formulate different management measures according to local conditions. Cities with higher GLUE levels should vigorously develop modern service industry and high-end manufacturing industry, introduce increasingly specialized human capital and knowledge capital into manufacturing industry through the form of service industry, ensure the continuity of manufacturing production and industrial upgrading. Backward cities with low GLUE levels should actively undertake the industrial transfer of advanced cities to promote the optimization of industrial structure. At the same time, build an information exchange platform between cities, break the barriers of information communication between regions, and improve GLUE. (3) Increasing the emphasis and publicity on sustainable development, paying more attention to the gradual transformation of resource-based industries, from high input and high energy consumption to low energy consumption and high value-added industries. Cultivating people's concept of sustainable development, popularizing low-carbon technology, developing circular economy, and accelerating the transformation of resource industry on the premise of resource conservation and environmental friendliness. Guiding people to pay attention to improve the efficiency of green investment, and rationally planning the scale of urban land. It is conducive to comprehensively improving the level of sustainable development of cities in an all-round way.

### Prospect

Resource-based cities are generally faced with the problem of transformation. The upgrading and rationalization of industrial structure conforms to the objective law of industrial transformation. On the basis of theoretical analysis, this paper uses the panel data of 115 resource-based cities from 2004 to 2018 and panel data model to empirically analyze the impact of industrial structure upgrading on GLUE, which enriches relevant research on the relationship between the two. However, there is room for further improvement in this study. Is there a U-shaped or inverted U-shaped nonlinear relationship between industrial structure upgrading and GLUE? Where is the inflection point between the two? Further empirical research is needed to answer these questions. In addition, the internal mechanism of industrial structure upgrading to improve GLUE needs to be further explored.

## Data Availability

The data that support the findings of this study are available from [www.cnki.net] but restrictions apply to the availability of these data, which were used under license for the current study, and so are not publicly available. Data are however available from the authors upon reasonable request and with permission of [www.cnki.net]. The datasets generated during and/or analysed during the current study are available from the corresponding author on reasonable request.
